# TNFα inhibitors as targets for protective therapies in MSA: a viewpoint

**DOI:** 10.1186/s12974-019-1477-5

**Published:** 2019-04-11

**Authors:** Alain Ndayisaba, Kurt Jellinger, Thomas Berger, Gregor K. Wenning

**Affiliations:** 1 Division of Clinical Neurobiology, Department of Neurology, Medical University of Innsbruck, Anichstraße 35, A-6020 Innsbruck, Austria; 2 Institute of Clinical Neurobiology, Alberichgasse 5/13, A-1150 Vienna, Austria; 3 Department of Neurology, Medical University of Vienna, Währinger Gürtel 18-20, A-1090 Vienna, Austria

**Keywords:** TNFα, TNFα inhibitors, Multiple system atrophy, Neuroinflammation, Neurodegeneration, Disease-modifying treatment

## Abstract

Multiple system atrophy (MSA) is a unique and fatal α-synucleinopathy associated with oligodendroglial inclusions and secondary neurodegeneration affecting striatum, substantia nigra, pons, and cerebellum. The pathogenesis remains elusive; however, there is emerging evidence suggesting a prominent role of neuroinflammation. Here, we critically review the relationship between αS and microglial activation depending on its aggregation state and its role in neuroinflammation to explore the potential of TNFα inhibitors as a treatment strategy for MSA and other neurodegenerative diseases.

## Background

Multiple system atrophy (MSA) is a rare, rapidly progressing and fatal neurodegenerative disease of unknown etiology that is clinically characterized by a variable combination of parkinsonism, cerebellar impairment, and autonomic and motor dysfunction [1, 2]. Together with Parkinson disease (PD) and dementia with Lewy bodies (DLB), MSA belongs to the neurodegenerative group of α-synucleinopathies which are characterized by the deposition of aberrant α-synuclein (αS) in both glial cells and neurons. MSA is considered a synucleinopathy with specific glioneural degeneration involving striatonigral, olivopontocerebellar, autonomic, and peripheral nervous systems [3, 4]. The neuropathological hallmark of this unique proteinopathy is the deposition of aberrant fibrillary αS in glial cells, mainly oligodendroglia, forming glial cytoplasmic inclusions (GCI) [5], which may even represent a primary pathologic event [3, 6, 7]. Less frequent are neuronal cytoplasmic inclusions (NCI) and other cellular deposits. Inclusion pathology is accompanied by neuronal loss, widespread demyelination, and gliosis. Degeneration of multiple neuronal pathways over the course of the disease causes a multifaceted clinical picture of this multisystem disorder [2]. The etiology and pathogenesis of MSA are not fully understood, but converging evidence suggests the propagation of misfolded αS from diseased neurons to oligodendroglia and its spreading from cell to cell in a “prion-like” manner [8, 9], inducing oxidative stress (OS), proteosomal and mitochondrial dysfunction, dysregulation of myelin lipids, decreased neurotrophic factor activity, neuroinflammation, and energy failure that result in a multisystem involvement [3, 4, 10–12]. Recent experimental and human studies demonstrated that deposition of αS and other pathologic proteins induces neuroinflammation not only in MSA but also in other neurodegenerative diseases, e.g., PD and Alzheimer disease (AD) [13–24]. In MSA, αS has been shown to mediate formation of abnormal inclusion bodies and to induce neuroinflammation, which, interestingly, may also favor the formation of intracellular αS aggregates as a consequence of cytokine release and the shift to a pro-inflammatory environment [23]. αS may directly activate microglia, and recent studies have shown that only fibrillary αS is an important inducer of pro-inflammatory immune responses [25], associated with increased production of key pro-inflammatory cytokines, like tumor necrosis factor (TNF)-α and interleukin-1β (IL-1β) [26]. The association of activated microglial cells and GCI burden [27] suggests that pathologic αS triggers inflammatory response in α-synucleinopathies by affecting αS aggregation and provoking cell death [28]. This was corroborated by a number of experimental studies in vitro and in vivo [29–31]. These and other studies supported the notion that microglial activation may contribute to the progression of the neurodegenerative process in MSA and in other synucleinopathies via increased levels of reactive oxygen species (ROS) [20, 32, 33], like in other neurodegenerative diseases [31]. Although this mechanism is non-specific, it may be exploited for therapeutic and neuroprotective interventions.

### TNFα in the central nervous system

TNFα, one of the key regulators in inflammation, belongs to the TNF ligand superfamily and is synthesized as a type II integral membrane protein occurring in a vast number of cell types. Within the central nervous system (CNS), microglia, astrocytes, and neurons are capable of synthesizing TNFα; however, activated microglia represent the main production site during neuroinflammatory processes [34, 35]. Following translation, it is synthesized as a transmembrane protein (tmTNFα) and cleavage by TNFα-converting enzyme (TACE) releases soluble TNFα (sTNFα). Both forms exert their functions on two receptors, TNFα receptor (TNFR) type I and II, with sTNFα preferentially binding to TNFR I, whereas tmTNFα has higher affinity towards TNFR II [36, 37]. The downstream signal-transduction cascades of TNFR I and TNFR II differ and imply the activation of numerous transcription factors including nuclear factor-kappa light chain enhancer of activated B cells (NF-κB) resulting in the regulation of various homeostatic and pathologic functions [38, 39]. In neurons, depending on the eventually activated transcription factor down the signaling pathway, TNFα drives either pro-apoptotic or pro-survival cell fate via TNFR I or TNFR II, respectively. Excessive release of TNFα, especially in a chronic manner as can be seen in many neurodegenerative diseases, leads to a shift towards receptor-independent neuronal cell death, directly through the activation of caspase 8 and 10 and indirectly by mediating glutamate excitotoxicity independent of receptor subtype. However, TNFR II also seems to exhibit neuroprotective properties as TNFR II has been shown to be critical for maintaining the oligodendrocyte progenitor pool in a cuprizone model of demyelination [40]. Furthermore, there is upcoming evidence on the role of TNFR II in oligodendroglial differentiation and thus remyelination capacity [41]. In addition, after knock-out of TNFR II, hippocampal neurons reveal increased sensitivity towards TNFα toxicity, whereas loss of TNFR I did not have this effect [42]. This raises the question whether inhibition of TACE and thereby largely depleting TNFR I activation while preserving tmTNFα triggered TNFR II signaling is to be considered in MSA, which will be discussed later on.

### TNFα in neurodegenerative disorders

Neuroinflammation characterized by microglial activation with secretion of many pro-inflammatory cytokines, in particular IL-1β and TNFα, has been implicated as main effector of the functional consequence of neurotoxicity, resulting in mitochondrial dysfunction [43], thereby contributing to the progress of neurodegeneration [13, 17, 44–47]. The two cytokines are potent mediators of microglial functions and modulate the complex networks of interactions of microglial-secreted molecules. The role of neuroinflammation has been demonstrated repeatedly in animal models of PD [21, 29, 48, 49] and of MSA [49–51]. The major pro-inflammatory cytokine released by activated microglia is TNFα secreted by the brain resident microglia/macrophages in response to various stimuli. Microglial TNFα plays a major role in angiotensin-induced dopaminergic cell death. Microglial release of TNFα is mediated by activation of angiotensin type 1 receptors, NADPH (nicotinamide adenine dinucleotide phosphate)-oxidase, Rho-kinase, and NFK-β [52]. An early increase in TNF, which leads to protein thiol oxidation resulting in activation of ASK1 (apoptosis signal-regulating kinase 1)-p38 signaling, may be critical for neuroprotection in PD [53]. Increased levels of TNFα and IL-1β have been detected in the cerebrospinal fluid of PD patients [54]; in serum of patients with MSA [55]; in p t-mortem tissue of DLB, PD, and MSA [56, 57]; and in animal models of PD [45, 58–62]. It has been demonstrated to play a major role in neuroinflammation-related cell death in PD, AD, and other CNS disorders. Experimental studies indicated that TNFα is toxic for dopaminergic neurons in vivo [63] and in vitro [64]. Long-term expression of TNFα seems to be necessary to exert univocal toxic effects in the substantia nigra (SN) [65]. The increased frequency of TNF1031, a high producer allele of TNF, in Japanese MSA patients compared with controls [66] and increase of TNFα rs1799964 and IL-1β rs16944 polymorphisms in Chinese patients with MSA [67] suggest that they may represent genetic risk factors for MSA and that TNF may have a toxic effect in MSA. Interleukin-8, intercellular adhesion molecule-1, and TNFα polymorphisms significantly increased the risk of MSA [68].

**Fig. 1:**
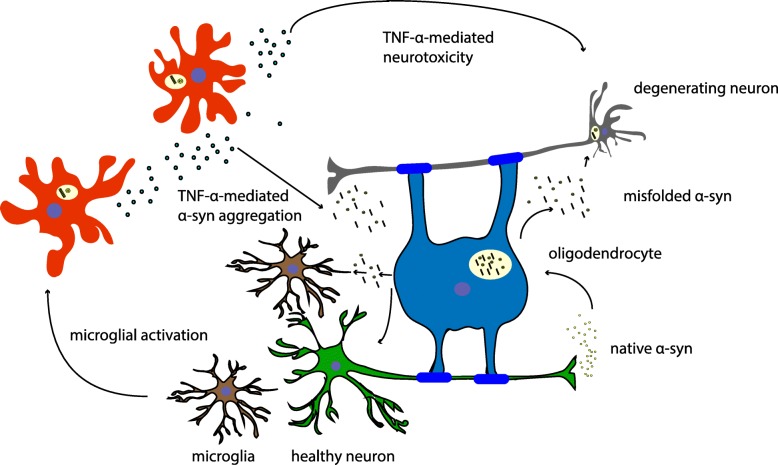
TNFα in MSA pathogenesis. Misfolded αS leads to microglial activation and subsequent microglial release of pro-inflammatory cytokines. High levels of TNFα in vulnerable brain areas in MSA contribute to αS aggregation and establish a pro-apoptotic environment in chronic disease

The importance of TNFα in the processes of development of PD is strengthened by animal models of PD, where elevated levels of TNFα are seen in a manner consistent with that of clinical PD [59, 61, 62]. Agents that interfere with TNFα synthesis and release seem to be protective in experimental models of PD [69, 70]. In other mouse models, conflicting findings regarding the protective effects of TNFα receptor deletion in toxin-induced PD have been reported [45, 64]. However, if lower levels of TNFα were expressed in the SN, a transient neuroprotective effect against 6-hydroxydopamine toxicity was observed [71]. On the other hand, when the synthesis of TNFα is unregulatory overproduced, this results in inappropriate cell death. TNFα and NF-κB expression and microglial activation in MSA [72] indicate an important role of TNFα in oligodendroglial cell death in this disease. High expression of TNFα in degenerating regions suggests that this potent pro-inflammatory cytokine is a mediator of neuronal injury. Furthermore, markers of inflammation such as serum T-lymphocyte-associated cytokine concentrations give evidence of immune mechanisms contributing to PD and MSA disease progression [73]. The pathobiological effects of TNFα in neurodegeneration and, in particular, in PD have been critically reviewed recently [17]. Figure 1 illustrates the effects of chronic TNFα-mediated neuroinflammation on oligodendroglia and neurons: TNFα drives oligodendroglial and neuronal cell death via activation of pro-apoptotic pathways and by increasing αS misfolding and aggregation.

Due to the possible cyclic nature of αS aggregation/release and gliosis in MSA, interventions that target neuroinflammation may have the potency to slow the progression of the disease and increase the quality of life [23]. Recent studies have approached α-synucleinopathies including MSA, by use of immunotherapy [74–76]. They have been shown to reduce αS co-localization in oligodendrocytes and astrocytes but increased microglial uptake, with decreasing demyelination, neuronal death, and motor deficit [76]. Other studies aimed at microglia as therapeutic target, e.g., treatment of the PLP-αS mouse model with minocycline reduced the density of activated microglia and inducible nitric oxidase synthase and toll-like receptor 4 immunoreactivity [51]. Recently, an inducer of heat shock proteins (HSP), carbenoxalone, was shown to decrease pro-inflammatory cytokines and OS in a rotenone model of PD [77]. Other treatments targeting neuroinflammation in glia and immune cells may be promising therapeutic strategies for MSA [23].

### TNFα inhibition as treatment strategy

Therapeutic regimes that interfere with either the synthesis of TNFα or the downregulation of TNFα/receptor interactions may have a considerable benefit to patients with such conditions. The use of agents similar to thalidomide and its analogs will inhibit TNFα protein biosynthesis, which will circumvent classical receptor subtype agonistic effects on signaling pathways. Furthermore, thalidomide will display biological activity in the CNS as it is a relatively small molecule and can cross the blood-brain barrier (BBB). Thus, it offers a better strategy for treating TNFα overproduction-induced disorders than that of currently available large proteins that bind and lower soluble TNFα [22]. Agents that interfere with pro-inflammatory events connected with TNFα synthesis and release have been shown to be protective in animal models of PD. Thalidomide has been shown to afford partial protection to striatal dopaminergic neurons in a 1-methyl-4-phenyl-1,2,3,6-tetrahydropyridine (MPTP) model [69, 70]. In transgenic mice, in which the gene for TNFα was functionally deleted, dopaminergic neurons in the striatum exhibited a greater degree of protection than wild-type littermates when challenged with MPTP [70]. However, in other mouse models employing gene targeting events on either one or both of the TNFα receptors (p55TNFR or p75TNFR), conflicting results regarding the protective effects of TNFα receptor deletion were reported [45, 64].

Thalidomide is widely known for its deleterious side effects since the late 50s/early 60s when birth defects were observed after pregnant women were prescribed thalidomide to treat morning sickness [78]. In addition, neuropathies may occur [79]. Meanwhile, however, thalidomide has been re-evaluated for immunomodulatory purposes as it enhances TNFα mRNA destabilization and degradation and, thereby, lowers its rate of synthesis and secretion [80, 81]. Furthermore, it is a co-stimulator of both CD8+ and CD4+ T cells [82], an inhibitor of angiogenesis [83] via its inhibitory actions on basic fibroblast growth factor (bFGF) and vascular endothelial growth factor (VEGF), and an inhibitor of the transcription factor NF-κB, whereas TNFα-induced leucine-rich α-2-glycoprotein-1 (LRG1) promotes angiogenesis and mesenchymal stem cell migration, this inhibition being a potential therapeutic approach [84].

Inhibition of the biological effects of sTNFα by etanercept and infliximab displayed beneficial properties against rheumatoid arthritis and other peripheral inflammatory diseases. Unfortunately, these agents are large macromolecules that minimally pass the BBB and thus will preclude their utility in CNS neurodegenerative disorders. BMS-561392 reduces the amount of sTNFα by inhibiting TACE; however, clinical phase II trials for rheumatoid arthritis have been halted due to mild hepatotoxicity [85], which might be due to the accumulation of tmTNFα and TNFRII increase. This effect has been described in available anti-TNF treatment, but in this case, hepatic injury usually takes a self-limiting course. In addition, BMS-561392 has shown poor ability to penetrate the BBB and can therefore not be considered a suitable candidate substrate. In contrast, thalidomide analogs can readily and rapidly pass through the BBB and, if well tolerated in animal studies, may be of potential in a wide spectrum of CNS diseases. The mechanisms underlying thalidomide’s actions have been summarized by [22]. In brief, the agent comprises two conjoined heterocyclic moieties—a phthalimide and a glutarimide ring showing structural modifications [86–89]. Recent studies confirmed that the actions of thiothalidomide agents were identical to that of thalidomide. They caused a concentration-dependent reduction in luciferase activity—consistent with the mechanism destabilizing the mRNA of TNFα [80], and thereby reduce TNFα synthesis. Thus, thalidomide and its analogs are excellent candidate agents for use in anti-TNFα therapies in a variety of diseases associated with neuroinflammation, particularly since they act at the levels of TNFα synthesis rather than for scavenge released protein or inhibit its interaction at a receptor level [22]. Infusion of mesenchymal stem cells in a transgenic mouse model of MSA inducing a downregulation of cytokines involved in neuroinflammation suggested a potent effect on immunomodulation and neuroprotection [90].

A recent study investigated the therapeutic efficacy of combining an unconventional anti-inflammatory therapy (lenalidomide, a small thalidomide derivative with immunomodulatory activity and therapeutic effects in multiple myeloma [91–93], with inhibition of TNFα production [93, 94]) with an αS-reducing immunotherapeutic approach (CD5-D5 single chain antibody) in a novel transgenic mouse model of MSA pathogenesis. The combined treatment achieved better results than each method alone; it reduced astro- and microgliosis, αS levels, and partially improved deficits in MBP (myelin basic protein)-αS transgenic mice. These effects were associated with an activation of the Akt signaling pathway, which may mediate cytoprotective effects downstream TNFα [75]. Other recent studies demonstrated the neuroprotective and anti-inflammatory activities of allyl isothiocyanate (AITC), an aliphatic isothiocyanate derived from the precursor sinigrin present in vegetables of the Brassica family, on microglial cells through attenuation of JNK/NF-κB/TNFα signaling, which may have significance in neurodegeneration [95]. Combined active humoral and cellular immunization approaches, which are capable of triggering neuroprotective responses of regulatory T cells (Tregs), support the further development of multifunctional (vaccine) approaches for the treatment of synucleinopathies [96]. These results open the door for the design of more complex clinical trials in which a carefully planned combination of therapeutic approaches can complement each other to target multiple aspects of the pathobiology and pathogenesis of MSA and related neurodegenerative disorders.

## Conclusions

TNFα-dependent neuroinflammation may play a key role in MSA pathogenesis, and its relevance has been underlined in various models of synucleinopathy. Targeting TNFα with readily available drugs may constitute a promising disease-modifying treatment in this hitherto incurable disease.

## Abbreviations

AD: Alzheimer disease
BBB: Blood-brain barrier
DLB: Dementia with Lewy bodies
GCI: Glial cytoplasmic inclusions
HSP: Heat shock proteins
IL-1β: Interleukin-1β
MPTP: 1-methyl-4-phenyl-1,2,3,6-tetrahydropyridine
MSA: Multiple system atrophy
NCI: Neuronal cytoplasmic inclusions
NF-κB: Nuclear factor-kappa light chain enhancer of activated B cells
OS: Oxidative stress
PD: Parkinson’s disease
ROS: Reactive oxygen species
SN: Substantia nigra
sTNFα: Soluble TNFα
TACE: Tumor necrosis factor α-converting enzyme
tmTNFα: Transmembrane tumor necrosis factor
TNFR: TNFα receptor
TNFα: Tumor necrosis factor α
αS: α-Synuclein
